# Efficacy of acceptance and commitment therapy on impulsivity and suicidality among clients with bipolar disorders: a randomized control trial

**DOI:** 10.1186/s12912-023-01443-1

**Published:** 2023-08-17

**Authors:** Mona Metwally El-Sayed, Eman Sameh Abd Elhay, Samah Mohamed Taha, Mahmoud Abdelwahab Khedr, Feby Saad Attalla Mansour, Ayman Mohamed El-Ashry

**Affiliations:** 1 Psychiatric and Mental Health Nursing, Faculty of Nursing, Alexandria University, Alexandria, Egypt; 2 Psychiatric and Mental Health Nursing, Faculty of Nursing, Mansoura University, Mansoura, Egypt; 3 Physiopsychology, Department of Psychology, Faculty of Arts, Alexandria University, Alexandria, Egypt

**Keywords:** Acceptance and commitment therapy, Impulsivity, Suicidality, Bipolar disorders

## Abstract

**Background:**

Among people with bipolar disorders, there are high rates of impulsivity and suicide attempts. Efforts to reduce suicide are hindered by the lack of conclusive evidence on interventional programs for those at risk. Thus, this work evaluated the efficacy of acceptance and commitment therapy on impulsivity and suicidality among bipolar clients.

**Methods:**

In a randomized controlled trial, 30 eligible clients with bipolar disorders were given Acceptance and Commitment Therapy, and 30 eligible clients for the control group were chosen randomly at a 1:1 ratio using Research Randomizer version 4.0. Clients completed the Acceptance and Action Questionnaire II, the Short Arabic Version of the Impulsivity Behavior Scale, and the Arabic Version of the Beck Scale for Suicide Ideation.

**Results:**

It can be observed that there was a statistically significant decrement in the mean scores of psychological inflexibility among the study group between baseline value (T0), posttest measurement (T1), and post-two-month follow-up (T2), from 32.91 SD (6.03) to 23.06 SD (6.22) post and 26.83 SD (3.49) post-two months, with an effect size of 0.846 (P < 0.001), compared to the control group, which revealed an increase in the mean score. The overall impulsivity among the study group between T0, T1, and T2 was 61.27 SD (4.57) to 46.83 SD (4.47) post- and 43.0 SD (5.30) post-two months, with an effect size of 0.906 (P < 0.001). Compared to the control group, which revealed a relative increase in the mean impulsivity score at the post- and post-two-month intervals, the Arabic Versions of the Beck Scale for Suicide Ideation Scale mean score before the intervention was 16.33 SD (6.08), then the post was 7.23 SD (4.72), and the post-two-month mean was 10.13 SD (5.49) with an effect size of 0.878 (P < 0.001) among the study group. On the other hand, mean scores of “suicide ideation” among clients in the control group increased posttest and nearly returned to the same value after two months.

**Conclusion:**

For bipolar clients suffering from suicidal thoughts and impulsive behaviors, acceptance and commitment therapy, an emerging third-wave behavior therapy, is an effective intervention.

**Trial registration:**

The study was registered retrospectively with reference number NCT05693389 on 23/1/2023, available at: https://clinicaltrials.gov/ct2/show/NCT05693389.

## Introduction

Suicide is a severe health problem, with a global mortality rate of 1.4% of all deaths. Annually, more than 700,000 people commit suicide [1]. One-half to two-thirds of all successfully completed suicides are caused by mood disorders. According to a meta-analysis, around 90% of suicide cases involved a mental illness, of which about 43.2% had some affective disorders, and 25.7% had problems with substance use [2]. About 30–40% and 50% of patients with affective disorders had major depressive disorder (MDD) and bipolar disorder (BD), respectively [3].

Bipolar disorder (BD), marked by recurrent manic/hypomanic and depressive episodes, has several subtypes, including bipolar I (BD-I), bipolar II (BD-II), and BD Not Otherwise Specified (BD-NOS) [4]. Bipolar disorder’s rapid cyclical nature, mixed episodes of agitated depression, early onset, comorbidity with anxiety disorders, and substance use disorders have all been identified as risk factors for suicide [5].

Suicidal behavior has a wide variety of complex causes. Although bipolar disorder (BD) is a significant trigger for suicide, suicidal behaviors cannot be fully explained by BD without the interaction of other factors, such as the severity of the illness, impulsivity, hopelessness, hostility, and aggression [6]. The research looked at impulsivity as either a trait characteristic of BD that remained constant throughout the disorder’s progression or as a state-dependent characteristic that fluctuated with the severity of the symptoms [7].

Impulsiveness is more likely to be observed in people with remitted BD. A general population study has shown that impulsivity predicts suicide attempts. The high rates of impulsivity in bipolar disorders are also linked to suicide attempts within that illness. However, a recent comprehensive meta-analysis of 70 trials revealed only minor impacts [8].

Nonetheless, in the scientific literature, the connection between impulsivity and suicidal behavior is well-established. It has been presumed that impulsivity facilitates the transition from suicidal ideation to a suicide attempt, and it has even been suggested that impulsivity is a more significant indicator of a suicide attempt than the presence of a specific suicide plan [9].

The absence of conclusive evidence on interventional programs targeted at the at-risk population is a critical limitation for reducing suicide and suicide attempts (e.g., clients with a suicide attempt history). In addition, the lack of randomized clinical studies limits our understanding of current therapy’s (RCTs) effectiveness. Although some therapies have been demonstrated to be effective, it has been challenging to implement and spread these programs in routine clinical practice [10].

Several studies have examined using cognitive behavior therapy (CBT) as a suicidality intervention; some findings indicate it is successful. Borderline personality disorder clients who report self-harm respond well to dialectical behavioral therapy (DBT) [11]. Additionally, acceptance and commitment therapy (ACT), known as the “third wave” of behavioral therapy, had positive effects on reducing suicidal ideation [12]. On the other hand, Morrison et al. (2020) [13] investigated the different diagnostic effects of ACT on impulsive decision-making and found that it can be utilized as a meta-diagnostic treatment for impulsive behaviors.

ACT may be helpful in the management of psychiatric diseases such as depressive episodes, eating disorders, borderline personality disorder, and psychosis, which are connected to an increased risk of suicide [14]. The foundation of ACT is the premise that people seeking treatment are encouraged to embrace painful feelings rather than try to suppress or modify them [15].

Moreover, ACT encourages psychological flexibility, acceptance of one’s own experiences, and dedication to behaviors consistent with one’s ideals. ACT therapies focus on six key processes to improve psychological flexibility: engagement with the present moment, acceptance, defusion, self as context, value clarification, and committed action [16, 17].

Therefore, we are dealing with a complicated, multifaceted, and multifactorial primarily psychological phenomenon marked by suffering and intolerable psychological pain, in which a person decides to end their life in a specific situation (insufferable, insoluble, interminable, inescapable, without a future or hope) [18]. Psychiatrists face a massive challenge in predicting and preventing suicidal behavior in their clients, but it may also be one of the most accurate measures of how well their clinical care works. In addition, high impulsivity scores are associated with increased overall functional impairment, a higher number of episodes with early onset, and a higher number of past suicide attempts, as well as increased substance intake [19].

Thus, this study evaluated the efficacy of acceptance and commitment therapy on psychological inflexibility, impulsivity, and suicidality among bipolar clients.

## Research hypothesizes

- Clients who engaged in acceptance and commitment therapy had less psychological inflexibility than the control group.
- Clients who engaged in acceptance and commitment therapy had less impulsivity than the control group.
- Clients who engaged in acceptance and commitment therapy had less suicidality than the control group.

## Methods and materials

### Study design

A randomized controlled trial was conducted between October 2022 and February 2023.

### Study setting

The study was conducted at the Main University Hospital’s psychiatric outpatient clinics affiliated with Alexandria University’s Faculty of Medicine. The clinics offer free treatment to clients with mental diseases. These treatments include mental health evaluation and diagnosis, pharmaceutical prescriptions, and counseling. The intervention took place in the outpatient clinic, which was equipped with various rooms to accommodate the different needs of the patients. The researchers chose a rehabilitation room with a quiet, welcoming, comfortable environment and a private and confidential space to demonstrate client therapy sessions. The clinics are open three days a week (Sunday, Monday, and Tuesday) for clients with mental illnesses from 8 a.m. to 2 p.m.

### Participants: sample size calculation and sampling technique

The number of clients with bipolar disorders who regularly visit psychiatric outpatient clinics ranges from one to four every day (216 to 864 clients/6 months), according to hospital statistics for 2021–2022. The sample size was determined using the G*Power Windows 3.1.9.7 program, with the following criteria: effect size = 0.25, err α prob = 0.05, power (1-err prob) = 0.85, number of groups examined = 2, and the number of measurements = 3, following Sim, and Lewis (2012) [20]. Thus, the study group contained 30 clients with a DSM-5 bipolar diagnosis, and the control group had a similar number.

### Inclusion criteria

Specific eligibility criteria were established to ensure participants’ suitability for the study. These criteria included the requirement that clients must be at least 18 years old, able to communicate coherently and meaningfully, possess reading and writing abilities, and not have an illness that has persisted for over 10 years. Their medical records were retrieved and reviewed to confirm the client’s eligibility. Outpatients who met the criteria for type I or II bipolar disorder as outlined in the Diagnostic and Statistical Manual for Mental Disorders, Fifth Edition (DSM-V) were selected as subjects for the study through a random selection process [4].

### Exclusion criteria

To ensure that bipolar disorders were not influenced by comorbid conditions, clients who exhibited chronic psychotic symptoms, were diagnosed with psychotic comorbidity, or were found under the influence of drugs or alcohol were excluded from participation in this study. The eligibility of potential participants was confirmed through a combination of direct questioning and reviewing their medical records.

### Random allocation

Using Research Randomizer version 4.0, eligible clients with BD were given ACT therapy at a 1:1 ratio chosen randomly. A software application generated random integers with a predefined group code. After getting written consent from eligible participants, a structured interview to investigate each client’s mood symptoms was conducted; the enrolling investigators asked the clients to choose a number between 1 and 60 to determine their group assignment. The trained researcher who prepared the software program and the enrolling investigators were not involved in any other trial operations; therefore, the allocation was kept secret. Throughout the trial, trained outcome assessors were blinded to group assignments. The ACT intervention will consist of eight sessions delivered over eight weeks, with evaluations of outcomes at baseline, study completion, and two-month follow-up.

**Fig. 1:**
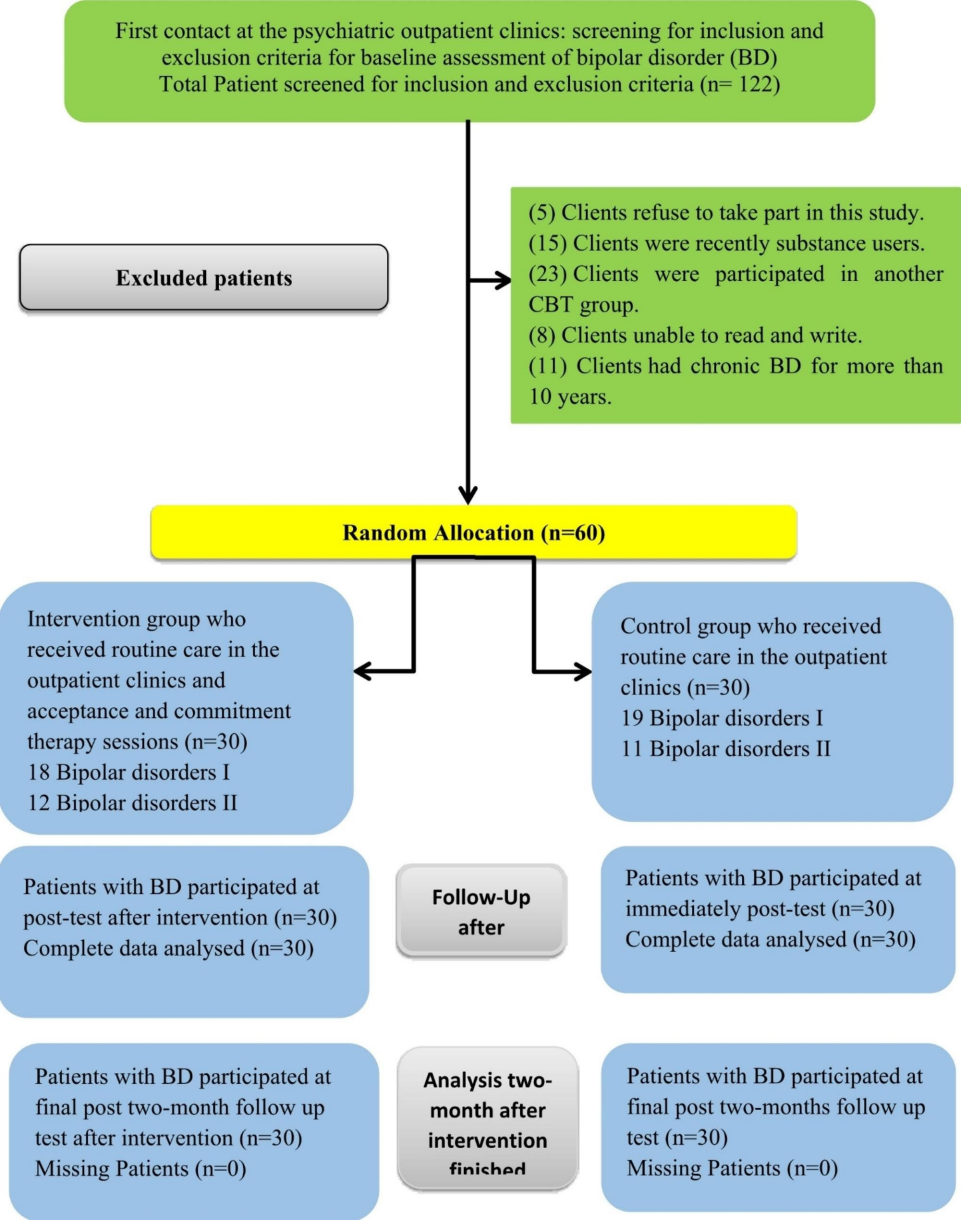
CONSORT flow diagram

The flow chart for BD (Fig. 1) shows that participants (n = 30) got acceptance and commitment therapy (ACT) face-to-face for 8 weeks. The immediate post-treatment assessments were performed with all BD clients in the intervention and control groups (n = 60). All participants completed the two-month follow-up posttest (30 from the intervention group and 30 from the control group).

### Tools for data collection

The data for the current study was gathered using the following tools:

#### Tool I: sociodemographic and clinical data, structured interview schedule

The researchers developed this tool to elicit data from clients’ sociodemographic characteristics, such as age and marital status. It was also used to collect information about the person’s level of education, the length of their first psychiatric visit, the length of their illness, the causes of previous episodes, and the medicines they were given.

#### Tool II: acceptance and action questionnaire-ii (AAQII)

**AAQII** consists of seven items on a Likert-type self-report scale to measure psychological flexibility [21]. Defusion, acceptance, and commitment to action are fundamental processes. Following each item is a Likert scale with the options never true [1] to always true [7]. Greater psychological inflexibility is indicated by higher scores, which fall within the 7–49 total score range, determined from the sum of the item responses. Cronbach’s alpha was 0.93, according to earlier studies [22]. The scale we employed had a standard Arabic translation, and Cronbach’s alpha was 0.81, indicating good internal consistency.

#### Tool III: the short Arabic version of the UPPS-P impulsivity behaviour scale

SUPPS-P consists of 20 items, initially developed by Whiteside & Lynam (2001) [23], to assess five aspects of impulsivity: Positive Urgency, Negative Urgency, Lack of Perseverance, Lack of Premeditation, and Sensation-Seeking. Every one of the five aspects is evaluated using a 4-point Likert scale. The Arabic version of SUPPS-P was first back-translated into Arabic by the writers Bteich, Berbiche, and Khazaal (2017) [24] after being professionally translated from French into Arabic. Based on Cronbach’s coefficients, the results showed that the scale was reliable, ranging from 0.58 to 0.81. Moreover, the authors use confirmatory factor analysis (CFA) to examine the covariance matrix. S-UPPS-P subscales showed statistically significant associations. Moreover, variations in the significance of the correlations ranged from.18 to.29, showing that the Arab short UPPS-P is a reliable evaluation tool with strong psychometric properties. In our study, the scale showed good internal consistency with α = 0.80.

#### Tool IV: the Arabic versions of the Beck scale for suicide ideation (BSSI)

Beck et al. (1979) developed the BSSI, a 19-item scale that measures how strong suicidal thoughts were the week before the test [25]. Beck et al. (1988) introduced the self-reporting edition of the measure. Each item is rated on an ordinal scale of 0 to 2; the total score ranges from 0 to 38 [26]. Individuals respond to the first five things that are excerpted. If an individual replies positively to the fifth item (scores 1 and 2), he or she answers the remaining items; otherwise, the questionnaire is finished.As a result, for data analysis, we used overall scale scores. With a Cronbach’s alpha range between 0.89 and 0.94 and a test-retest coefficient of 0.79, we used the study of Alsalman & Alansari (2019) to demonstrate satisfactory reliability [27]. Also, the internal consistency of the scale in this study reveals its reliability with α = 0.82.

### Procedure

#### Ethics approval and consent to participate

The Ethical Committee of Alexandria University’s Faculty of Nursing reviewed and accepted the study proposal. An agreement from the head of the psychiatric outpatient clinics at the Main Alexandria University Hospital for Psychiatric Medicine and the Patients’ Rights Protection Committee of the Ministry of Health and Population’s General Secretariat of Mental Health in Cairo was obtained to conduct the trial. Following an explanation of the therapy’s aims, each client participating in the therapy provided informed written permission. It was protected by keeping the client’s identity and personal data confidential. The client’s right to decline or withdraw from the research at any moment was underlined.

**Preparation: A** structured interview took 10 to 15 min to collect the baseline assessment data using the study scales SUPPS-P and BSSI.

**Pilot study**: In this study, five clients with BD were chosen randomly to determine clarity and any barriers encountered during data collection; those individuals were eliminated from the main study. The instruments were clear, intelligible, and valuable in the pilot study. The Alpha Cronbach’s test was used to determine the internal consistency of the study instrument.

**Intervention phase**: The ACT intervention consists of five groups, each with six clients [28, 29]. The ACT sessions were 90 min long and held weekly for eight weeks. During ACT, all clients continued their usual care (TAU), including receiving one or more mood stabilizers prescribed by their usual psychiatrist, expert care coordination, and emotional support. ACT therapy group sessions were face-to-face with two certified ACT psychiatric nurse researchers for five consecutive groups to design a framework for each session and, at the same time, work through exercises in response to the direct experiences of each group. The sessions comprised two psychoeducational sessions and six skill-training sessions in which clients acquired skills (see Table 1 for the session time plan).

**Table 1:** Time Plan of the Session

Session time plan (90 min)	• 5 min: practicing mindfulness, examining the self and the external world. • 15 min: Review previous concepts and challenges encountered by BD clients during skill demonstration. • 30 min: The trained researcher will apply and demonstrate the session’s new concepts through videos. • 30 min: Discussed the session’s objective and skills with the clients and asked them to demonstrate the strategies they learned throughout the session. • 10 min: homework assignments.

**Phase I** consisted of two sessions, with the first focusing on the ACT session’s general goals and expectations of participants. The second session concentrated on psychoeducation; topics concerning drugs, symptoms, and the history of the illness were thoroughly addressed to equip clients with the essential information required to comprehend BD. Based on a treatment manual and applications of ACT developed by Hayes et al. (1999), Bach & Hayes (2002), Bloy, Oliver, & Morris (2011), El-Ashry et al. (2021) [16, 30–32] on clients with psychosis, and O’Donoghue et al. (2018) on clients with BD, the researchers created a psychoeducational module of the ACT strategy [28].

**In Phase II**, clients attended six skill-training sessions to learn ACT and therapy skills. After cultural adaptation to the Egyptian context, a structured manual was given to the clients. So, each session’s main aims and materials were translated into Arabic. Accordingly, each ACT session’s general and specific goals were established. According to the ACT approach, the goal of the intervention was to help the person become more psychologically flexible (have a more accepting, thoughtful, and calm response to BD symptoms and related emotions and thoughts) so that they could act in line with their values. The ACT intervention emphasizes psychoeducational methods for bipolar and mood disorders. Each session focuses on a unique aspect of bipolar disorders, such as mania, stress, impulsivity, sadness, medication, talking with loved ones, and suicide. Each session presents ACT techniques as an alternative response to bipolar disorder symptoms. The ACT groups are meant to be collaborative, involving experiential activities throughout, a commitment to working towards a valued goal, and mindfulness exercises between sessions.

The intervention’s exercises (such as brief mindfulness, defusion, and value clarification) highlight how participants may accidentally become “caught up” in struggling with their BD symptoms and distress and thus adopt maladaptive coping strategies such as impulsivity and suicide. Activities were brief, and learning points were timed and structured to meet challenges. Particular emphasis was placed on encouraging practice at home. A prepared film of an actor portraying a man expressing obstacles in his life was used to demonstrate the metaphor’s real-world relevance and broad applicability. We recognize that eight weeks is a relatively brief period for evaluating change. This period is adequate for the practical aims of this study, as we are more concerned with the possible usefulness of the intervention than with its lasting consequences (see Table 2).

**Table 2:** ACT intervention Sessions for Clients with Bipolar Disorders

Session	Objective	Metaphors	Content
Psychoeducation – Overview of ACT expectations of participants	To provide clients with an overview of ACT. ACT expectations of participants.	Describing the expectations from the therapy.	• The researchers explained the ACT session’s general goals • The researchers demonstrated ACT expectations of participants
Psychoeducation – Discussion of a booklet and videotape about BD.	• To be conscious about symptoms, drugs, and the history of the BD illness • Topics concerning drugs, symptoms, and the history of the illness were thoroughly addressed to equip clients with the fundamental information required to comprehend BD	videotape about BD	• The researchers demonstrated BD symptoms, drugs, and the history of the illness were thoroughly addressed to equip clients with the essential information required to comprehend BD.
Session 1 Learning to be Present: How to Feel and Be Here and Now	• To be conscious of what is going on around you. • To be aware of one’s thoughts, feelings, and body sensations (i.e., private experience). • To differentiate between what is present on the inside and what is present on the outside, and to explain these processes without making a judgment.	• Noticing what’s Outside: The World, You and Me. • Noticing What’s Inside: Thoughts, Feelings, and Physical Sensations. • Why I Can’t Be Here—What’s Distracting? • Noticing the room. • Describing the Self and Another. • The bag and card. • The tug of war with monster images.	• The researchers demonstrated how vital features of ACT contribute to psychological flexibility and cognitive defusion while dealing with impulsivity and suicidal thoughts. • The clients were taught to focus on their surroundings rather than their inner experiences by the researchers (such as thoughts, feelings, and bodily sensations). • The researchers encouraged the client to look at his habitual emotional control agenda as a response to delusional ideas and their influence on his life. • The choice point model was employed by the researchers to express this meaning and emphasize acceptance as a useful coping tool. Discuss the idea of creative hopelessness in relation to the traditional emotional control agenda. • The researchers advise the client to set aside the negative ideas and concentrate on willingness. • These skills lead to self-development: “I am feeling….“ “I am thinking….“
Session 2 Defusing impulsivity and suicidal thoughts	• Introduces the concept of the mind and how it generates linguistic processes (thoughts and delusions). • To be able to distinguish between ideas and other internal feelings.	• Addressing the Verbal Network in the Mind. • Experiences and Thoughts Training Your Co-Pilot • Thoughts about the Bell Listening to Your Co-pilot: The Thoughts He Gives Me • Passengers on Your Plane.	• The researchers employed mindfulness techniques to help the client connect with the present moment and develop psychological flexibility to deal with impulsivity and suicidal thoughts. • The researchers suggested that the client utilize the language of noticing when referring to behaviour altered due to internal experiences of negative thoughts or other thoughts and emotions triggered throughout the session, such as “I’m noting that…” • Using the metaphor “Passenger on Your Plane,“ the researchers made a video to explain to the client how delusional ideas come and fade from the standpoint of an observer.
Session 3 A Review of Acceptance: Goals, Purpose, and Process Suggestions	• To acquaint clients with the sensation of trying to force unwanted thoughts and feelings away (i.e., to identify the process of experiential avoidance). • To demonstrate the inefficiency and ineffectiveness of experiencing avoidance to the client. • To encourage acceptance as an alternative to personal experience-based avoidance methods.	• Unwanted Thoughts • Unwanted Feelings • Dropping the Rope—Willingness • Getting Rid of Thoughts • What is Unwanted? • Meeting One Another’s Co-Pilots • Standing with Yourself • Dropping the Rope	• The decision point model changed to illustrate how suicidal ideas and sources of fusion are related, as well as how they affect moving away from the life the client wishes to live. • The researchers instructed the client to perform exercises that distanced him from the imagined self in order to build a separation between them. The client was asked to repeat all distance exercises, including “I am having the thought that…” and “identifying your thoughts.“
Session 4 A Review of Self as Context: Goals, Purpose, and Process Suggestions	• For clients to become aware of the self as content (content you), and that the “content you” (thoughts of, “I like ______, I am a ______,“ etc.) • Changes over time and situations as do feelings, physical sensations, and other thoughts—they are impermanent. • For clients to become aware of the self as constant (constant you) and that the “constant you” does not change over time—it is permanent.	• I Am the Person Who… • Who is the “You” who notices? • The “Constant You” • Noticing & Sharing Who I am • Acting 101 • Wisdom with Age—Images of Youth. • I Have Survived	• By monitoring suicidal and negative thoughts on many occasions, the researchers were able to assist the client comprehend the various aspects of self-conceptualization. • The researchers focused the client’s attention to the contrast between the self who sees such thoughts and the negative thoughts that occur. • The researcher employed the metaphor of leaves on a stream by presenting a film and asking the client to see their thoughts and feelings as leaves flowing down a stream without trying to halt or control them. • The contrast between the outcomes and components of self-evaluations and the self that assesses was developed and emphasized by the researcher. In order to illustrate the distinction between ideas and self, the researcher employed the metaphor of a chessboard while playing the game with the client.
Session 5 Clarification of Personal Values Goals, Purpose, and Process Suggestions	• Identify meaningful, valued domains of life. • Define specific behavioural goals that align with those values • Identify potential barriers to following through with valued-based living	• Where Do I Want to Fly? • Defining My Next Destination • Exploring New Terrain • Flying South • Choosing a Direction • Goal Sharing • Initial Discussion Regarding Values Clarification • Chain Analyses of Client Behaviour	• The researchers spoke with the client about how prior attempts to fight unpleasant thoughts impact and get in the way of achieving his goals and, in turn, his values. • The researcher assisted the client in considering potential values that could fulfil his life purpose. • The client’s values were clarified and categorized with the use of a Bull’s Eye worksheet by the researchers. • The researchers used the compass metaphor to explain to the client how values relate to goals and the distinction between value and goal. • The disparity between his prized area and his everyday activities as a result of his negative thoughts was the focus of the researchers’ attention. • By using a worksheet based on the client’s selected value from the Bull’s eye, the researchers were able to assist the client in setting a SMART goal and encouraging him to act on his value rather than on irrational or unpleasant perspectives.
Session 6 Moving Ahead with Committed Action Goals, Purpose, and Process Suggestions	• To continue clarification of value-based goals. • To identify barriers to committed action. • To increase committed action in goal-completion, building patterns of committed action. • To increase the generalization of skills for valued living, with the intention of continued committed action upon completion of the protocol.	• Turbulence in Flight: What Makes for a Rough Ride? • Willingness in Action Committed to a Valued Path • Flying in a Meaningful Direction with Turbulence: Are You Willing? • Commitment Statements	• The researchers concentrated on values that were established using the Bully’s eye. • The researchers have started to create an action plan for one of the value domains from the Bully’s eye. • The client made a list of specific activities (such as reading the Quran, praying, drawing, conversing with other individuals, practicing mindful walking in the garden, and engaging in some physical exercise) that are pertinent to his short-term goals and consistent with the selected value, ranking them according to how challenging they are to complete. • The researchers kept track of any obstacles that may stand in the way of his attaining his objectives as well as how he handled them when they did. • “Flying in a Meaningful Direction with Turbulence: Are You Willing?“ was the subject of a worksheet and video presented by the researchers. This is a metaphor used to persuade the client to remain committed to the action plans despite obstacles. • The client’s opinion on the ACT sessions was then asked out.

**The posttest phase** was performed twice immediately following the end of all sessions and a two-month follow-up using S-UPPS-P and BSSI.

### The control group

As a control group, clients were randomly assigned to their respective groups and continued to receive their usual care (TAU), which included receiving one or more mood stabilizers and antipsychotics as recommended by their psychiatrist from the outpatient clinic. Assessments and evaluations were conducted concurrently with the intervention group on the screening day and two months later at the end of the study. It was worth mentioning that the control group was on a waiting list, as they received the same intervention as the study group after the trial finished. The researchers contacted the clients through their phones and What’s Up groups.

### Data processing and analysis

Data were put into the computer, and the IBM SPSS software package, version 23.0, was used to analyze the data. To compare two categories of normally distributed quantitative variables, the Student t-test was used. ANOVA with repeated measures followed by Bonferroni adjustment for multiple comparisons between the three periods in each group. The Shapiro-Wilk test was used to verify the variable distribution’s normality. The Chi-square test is used when categorical variables are compared between groups (Monte Carlo or Fisher Exact). A 5% level of significance was applied to the obtained results.

## Results

The study found that most clients in the intervention group (56.7%) and the control group (40%) were between 30 and 39. Over half of the clients in both groups (63.3% in the intervention group and 56.7% in the control group) held a university degree. Single clients accounted for 56.7% of the intervention group and 46.7% of the control group. More than half of the clients in both groups had families with 2 to 4 members (53%). Additionally, most clients in both groups (63.3% in the intervention group and 53.3% in the control group) considered their income sufficient (Table 3).

**Table 3:** Distribution of the studied cases regarding their sociodemographic data characteristics

Sociodemographic and clinical characteristics.	Intervention group (n = 30)		Control group (n = 30)		χ^2^	p
	**N**	**%**	**N**	**%**		
**Age**						
20–29	8	26.7%	9	30.0%	2.144	^**MC**^**p** = 0.574
30–39	17	56.7%	12	40.0%		
40–49	4	13.3%	7	23.3%		
50-	1	3.3%	2	6.7%		
**Sex**						
Female	18	60.0%	12	40.0%	2.400	0.121
Male	12	40.0%	18	60.0%		
**Education**						
Secondary	6	20.0%	9	30.0%	0.867	^**MC**^**p** = 0.766
University	19	63.3%	17	56.7%		
Postgraduate	5	16.7%	4	13.3%		
**Occupation**						
Employed	15	50.0%	15	50.0%	0.994	^**MC**^**p** = 1.000
Unemployed	14	46.7%	15	50.0%		
Retired	1	3.3%	0	0.0%		
**Marital status**						
Single	17	56.7%	14	46.7%	1.207	^**MC**^**p** = 0.663
Married	9	30.0%	13	43.3%		
Divorced	4	13.3%	3	10.0%		
**Family no**						
2–4	16	53.3%	16	53.3%	0.477	^**MC**^**p** = 1.000
5–7	12	40.0%	13	43.3%		
> 7	2	6.7%	1	3.3%		
**Income**						
Enough	19	63.3%	16	53.3%	0.617	0.432
in enough	11	36.7%	14	46.7%		

χ^2^: Chi-square test MC: Monte Carlo.

In the intervention group, 36.7% of clients experienced bipolar disorder symptoms for less than a year, while only 20% were in the control group. More than half of the participants in both groups had been hospitalized one to five times (76.7% in the study group and 53.3% in the control group). Most clients in both groups reported no physical illnesses (70% in the intervention group and 60% in the control group). Treatment methods included ECT sessions for 30% of participants in both groups and pharmaceutical treatment for 70%. There were no significant differences in sociodemographic and clinical characteristics between the intervention and control groups, indicating they were well-matched (see Table 4).

**Table 4:** Distribution of the studied cases regarding their clinical data

Clinical data	Intervention group (n = 30)		Control group (n = 30)		χ^2^	p
	**N**	**%**	**N**	**%**		
**The onset of the disorder**						
less than one year	11	36.7%	6	20.0%	5.632	^**MC**^**p** = 0.138
1–5	7	23.3%	11	36.7%		
5–9	6	20.0%	2	6.7%		
10–20	6	20.0%	11	36.7%		
**No of admission**						
1–5	23	76.7%	16	53.3%	4.991	^**MC**^**p** = 0.096
6–10	3	10.0%	10	33.3%		
11–20	4	13.3%	4	13.3%		
**Physical illness**						
Yes	9	30.0%	12	40.0%	0.659	0.417
No	21	70.0%	18	60.0%		
**Treatment**						
Medications	21	70.0%	21	70.0%	0.0	1.000
ECT	9	30.0%	9	30.0%		
**Family history**						
Yes	16	53.3%	20	66.7%	1.111	0.292
No	14	46.7%	10	33.3%		

χ^2^: Chi-square test MC: Monte Carlo.

The baseline mean score of the AAQ-II was 31.81 (SD = 6.08) for the intervention group and 32.73 (SD = 4.98) for the control group. After the intervention, the intervention group’s mean AAQ-II score decreased significantly to 22.05 (SD = 5.72), while the control group’s mean score increased slightly to 33.43 (SD = 5.26). The difference between the two groups was significant, with a large effect size (t = 6.020, p < 0.001, η2 = 0.856). At the two-month follow-up, the intervention group’s mean AAQ-II score remained lower than the pre-intervention score at 24.13 (SD = 4.49), while the control group’s mean score decreased slightly to 31.21 (SD = 4.31). The difference between the two groups was significant with a medium effect size (t = 3.124, p = 0.014, η2 = 0.314) (see Table 5).

**Table 5:** Description of mean scores and standard deviations of Acceptance and Action Questionnaire-II (AAQ-II)) among the Intervention and control groups at pre, post, and post two-month of the ACT intervention

Acceptance and Action Questionnaire-II**	Intervention group (n = 30)		Control group (n = 30)		t (p)
	M	SD	M	SD	
Pre	31.81	6.08	32.73	4.98	0.679 (0.536)
post	22.05	5.72	33.43	5.26	6.020* (< 0.001*)
Post two-month	24.13	4.49	31.21	4.31	3.124* (0.014*)
F (P) η^2^	73.321* < 0.001* 0.856		2.431 0.190 0.314		

SD: Standard Deviation t = independent t-test F = ANOVA with repeated measures η2 = Partial Eta Squire * statistically significant p-value at ≤ 0.05.

**the more mean score reflected more in psychological inflexibility.

There were no significant differences between the intervention and control groups regarding subscales or the overall S-UPPS-P score at the baseline assessment. However, after the intervention sessions, the intervention group demonstrated significant reductions in positive eagerness, negative urgency, lack of perseverance, lack of premeditation, and sensation seeking. On the other hand, the control group did not show any significant changes in subscales or the overall S-UPPS-P score. The differences between the intervention and control groups were statistically significant for all subscales and the overall S-UPPS-P score, with large effect sizes (all p < 0.001, η2 = 0.945). The intervention group maintained significant reductions in all subscales and the overall S-UPPS-P score even after a two-month follow-up, while the control group did not exhibit any significant changes (see Table 6).

**Table 6:** Description of mean scores and standard deviations of The Arab S-UPPS-P impulsivity scale among the intervention and control groups at pre, post, and post two- month of the ACT intervention

The Arab S-UPPS-P impulsivity scale			Intervention group (n = 30)				Control group(n = 30)				T (p)
			M		SD		M			SD	
Positive eagerness	**Pre**		11.67		1.84		11.47			3.40	0.283 (0.778)
	**Post**		9.30		2.49		12.70			3.98	3.962* (< 0.001*)
	**Post two-month**		8.27		2.41		11.73			2.68	5.275* (< 0.001*)
F (P) η^2^			22.053* < 0.001* 0.611				1.003 0.373 0.049				
Negative urgency	**Pre**		12.93		1.66		13.37			3.20	0.658 (0.513)
	**Post**		10.23		1.57		12.77			3.89	3.306* (0.002*)
	**Post two-month**		9.0		2.15		12.13			2.57	5.123* (< 0.001*)
F (P) η^2^			50.840* < 0.001* 0.668				1.275 0.287 0.103				
Lack of perseverance	**Pre**		10.97		2.79		10.40			1.85	0.928 (0.357)
	**Post**		9.17		2.48		10.87			1.63	3.136* (0.003*)
	**Post two-month**		8.67		1.73		10.87			1.91	4.681* (< 0.001*)
F (P) η^2^			29.088* < 0.001* 0.577				2.355 0.104 0.119				
Lack of premeditation	**Pre**		12.67		2.20		11.50			2.76	1.808 (0.076)
	**Post**		7.30		1.73		11.80			1.86	9.704* (< 0.001*)
	**Post two-month**		7.0		1.80		11.60			2.22	8.810* (< 0.001*)
F (P) η^2^			276.512* < 0.001* 0.945				0.372 0.691 0.026				
Sensation seeking	**Pre**		13.03		1.83		12.97			3.35	0.096 (0.924)
	**Post**		10.83		1.42		13.57			3.58	3.890* (< 0.001*)
	**Post two-month**		10.07		2.77		13.17			3.05	4.122* (< 0.001*)
F (P) η2			16.437* < 0.001* 0.560				0.388 0.680 0.013				
Overall S-UPPS-P	**Pre**		61.27		4.57		59.70			7.66	0.962 (0.340)
	**Post**		46.83		4.47		61.70			7.35	9.469* (< 0.001*)
	**Post two-month**		43.0		5.30		59.93			6.40	11.158* (< 0.001*)
F (P) η2			181.569* < 0.001* 0.906				1.090 0.343 0.058				

SD: Standard Deviation t = independent t-test F = ANOVA with repeated measures η2 = Partial Eta Squire * statistically significant p-value at ≤ 0.05.

No significant differences were observed between the intervention and control groups regarding their BSI scores at the baseline assessment. However, after the ACT sessions, the intervention group demonstrated a significant reduction in BSI scores, with a mean score of 7.23 (SD = 4.72), compared to the control group’s mean score of 14.43 (SD = 4.26), at the post-intervention assessment. The difference between the two groups was statistically significant (T = 6.196, p < 0.001), with a large effect size (η2 = 0.878). Additionally, the intervention group maintained a significant reduction in BSI scores even after a two-month follow-up assessment, with a mean score of 10.13 (SD = 5.49), compared to the control group’s mean score of 13.37 (SD = 4.31). The difference between the two groups remained statistically significant (T = 2.536, p = 0.014), with a medium effect size (η2 = 0.344) (see Table 7).

**Table 7:** Description of mean scores and SD of The Arabic versions of the Beck Scale for Suicide Ideation (BSI) among the intervention and control groups at pre, post and post two-month of the ACT intervention

Beck scale for Suicide ideation		Intervention group (n = 30)		Control group (n = 30)		T (p)
		M	SD	M	SD	
Total (BSI)	**Pre**	16.33	6.08	13.73	3.98	1.809 (0.076)
	**Post**	7.23	4.72	14.43	4.26	6.196* (< 0.001*)
	**Post two-month**	10.13	5.49	13.37	4.31	2.536* (0.014*)
F (P) η^2^		70.321* < 0.001* 0.878		4.222* 0.019* 0.344		

SD: Standard Deviation t = independent t-test F = ANOVA with repeated measures η2 = Partial Eta Squire * statistically significant p-value at ≤ 0.05.

## Discussion

This randomized controlled trial was one of the few studies examining ACT therapy’s effectiveness in reducing suicidal ideation in people with bipolar disorders. According to prior research, clients with bipolar type 1 are more likely to be impulsive. Unexpectedly, impulsivity is a prominent trait of eurythmics, and it does not only appear during manic or depressive episodes [5]. Impulsivity is linked to increased functional impairment, higher hospitalization rates, a higher risk of suicide, and illness severity in BP [33]. Thus, we also investigated the effectiveness of ACT therapy on impulsivity as a secondary outcome.

According to our research, compared to standard of care alone, ACT therapy significantly reduced suicidal thoughts in bipolar clients and improved impulse control. It can be explained that ACT therapy primarily focuses on experiential avoidance, the propensity to suppress unwanted thoughts or emotions to increase psychological flexibility. It helps clients find hope, cultivate a fulfilling life, and learn mindfulness.

Clients with bipolar disorders in the intervention group also improved their psychological flexibility regarding their impulsive acts and suicidal thoughts compared to the control group. That was consistent with a study by Pankowski et al. (2017), who applied group acceptance and commitment therapy (ACT) for bipolar disorders; they reported a highly significant improvement in psychological flexibility among the intervention groups with a large effect size. The ACT intervention focused primarily on improving clients’ responses to complex thoughts and feelings by modifying their relationships with them [29].

One of the primary areas of ACT treatment is escape, also known as experiential avoidance. Some of the most common psychological frameworks used to explain suicide, particularly the entrapment/cry of pain model, include pain escape as a critical factor [34]. Mindfulness skills, acceptance of distress, and defusion from distressing thoughts can all help an individual cope with the discomfort of severe emotional pain. Finally, identifying personal values and engaging in positive action aligned with these values may result in a more integrated individual, thereby improving well-being [11].

Similarly, Ducasse et al. (2018) [14], in a randomized controlled trial comparing a 7-week relaxation group versus acceptance and commitment therapy as an add-on to standard care for adult outpatients with a current suicidal behavior disorder, found that at the post-therapy assessment, the rate of change in ACT for suicidal ideation was higher than in the relaxation group [12]. In a quasi-experimental study, Ghadam et al. (2020) examined the impact of ACT therapy on reducing suicidal ideation in addition to anxiety and depressive symptoms. They discovered that ACT significantly helped manage and reduce suicide ideation, anxiety symptoms, and depressive symptoms in the experimental group [15]. Najafi & Arab (2020) reported that ACT Therapy can improve psychological capital and emotion regulation in people with suicidal ideation, so it is recommended to reduce suicidal thoughts [35]. Conversely, Torok et al. (2020) confirmed that studies that directly addressed suicide showed the most significant reductions in suicidal ideation, whereas indirect interventions aimed at treating depression did not [36]. After reviewing 44 studies, Meerwijk and colleagues concluded that indirect interventions had no appreciable short-term impact on suicide [37].

Our results were in line with Polat & Karakaş (2021), who conducted a randomized control trial to examine the impact of ACT therapy-oriented anger management training on anger rumination and impulsivity levels in individuals receiving forensic mental healthcare. They discovered that the experimental group’s total scores on the Barratt Impulsiveness and Anger Rumination Scales were significantly lower than those of the control group [38]. In this context, Baghani & Akbari (2020) confirmed that cognitive defusion, the effect of thought (such as the desire to use) on behavior in acceptance and commitment therapy, can explain why ACT therapy effectively reduces the impulsivity components of women who abuse drugs. Behavior dependent on context or thought is categorized as cognitive fusion or cognitive defusion, and when a person is fused with their thoughts, they cannot discriminate between their mental judgment and reality [39]. Hasani, Hasani, & Niaei (2019) reported that through increased awareness, psychological acceptance, emotional isolation, emotion management, a reduction in ineffective control, and problem-solving techniques, ACT therapy lessens the impulsivity of clients with bipolar disorders and major depressive disorder [40]. Morrison et al. (2020) investigated the efficacy of ACT therapy in impulsive decision-making. The findings indicated that this therapy increased productivity and flexibility in emotions, thoughts, and physical states and that the ACT was a valuable therapy for helping people change their behavior and improve their quality of life [13].

**Hayes et al. (2012)** explained that ACT therapy helps clients accept their uncontrolled emotions and cognitions and be spared from regulating the linguistic rules that have caused those troubles rather than removing damaging variables. This treatment strategy aims to lessen impulsivity by fostering psychological flexibility on the one hand and acting on personal values on the other, and it assists clients in building an entire, meaningful life [41]. Developing a self-observer in clients through defusion skills, mindfulness, and present-moment awareness is another aspect of this therapy that aids in reducing impulsivity. Mindfulness can help increase awareness of automatic thoughts, enhancing a person’s ability to assess the potential implications of actions before engaging in them [42].

### Limitations

The study’s follow-up period only lasted two months after the intervention, which means that any long-term effects of the treatment could not be evaluated. Additionally, cultural differences may exist in understanding concepts such as acceptance, defusion, and metaphors, which should be considered when interpreting and generalizing the study’s results. Therefore, caution should be exercised when drawing broader conclusions from the study. As the small sample size was a significant limitation and could affect the study’s statistical power and generalizability, it was recommended to do other studies with large sample sizes.

## Conclusions

Even though our study sheds light on the efficacy of ACT on impulsivity and suicidality among clients with bipolar disorders in a specific ethnocultural group, due to the small sample size and cultural specificity, care should be taken when generalizing our results to other populations. To replicate and build on our findings and investigate the significance of culturally specific elements in the connection between impulsivity and suicidal behavior, additional research with more extensive and diverse samples is required. The ACT intervention is an effective interventional strategy for bipolar disorder clients to lessen suicidal thoughts and impulsive behaviors, so it is essential and apparent that the fundamental ACT principles be applied to these clients.

### Nursing implications

In outpatient clinics, ACT must be a more prominent intervention for at-risk clients with BD who make impulsive decisions and suffer recurrent suicidal thoughts. Therefore, ACT may lessen the severity and/or frequency of suicidal ideations through several mechanisms, including improving acceptance skills due to a shift in the client’s connection to their internal experiences and clarity of what is significant in their lives. Life becomes more meaningful through increased personal commitment to behaviors that uphold values. A negative effect on modifiable suicide risk variables (impulsivity, hopelessness, and psychological suffering).

## Data Availability

On reasonable request, data will be available from the corresponding author (ayman.el-ashry@alexu.edu.eg).

## Abbreviations

ACT: Acceptance and Commitment Therapy
BD: Bipolar Disorders
BSI: Beck Scale for Suicide Ideation
RCT: Randomized Control Trial
S-UPPUS-P: Short Arabic Version of the UPPS-P Impulsivity Behavior
TAU: Treatment as Usual
